# Metabolic Dysfunction-Associated Fatty Liver Disease Is Associated with the Risk of Incident Cardiovascular Disease: A Prospective Cohort Study in Xinjiang

**DOI:** 10.3390/nu14122361

**Published:** 2022-06-07

**Authors:** Yanbo Guo, Jing Yang, Rulin Ma, Xianghui Zhang, Heng Guo, Jia He, Xinping Wang, Boyu Cao, Remina Maimaitijiang, Yu Li, Xinyu Peng, Shijie Zhang, Shuxia Guo

**Affiliations:** 1 Department of Public Health, Shihezi University School of Medicine, Shihezi 832000, China; guoyanbo@stu.shzu.edu.cn (Y.G.); marulin@shzu.edu.cn (R.M.); michaelzhang818@163.com (X.Z.); guoheng@shzu.edu.cn (H.G.); hejia123.shihezi@163.com (J.H.); wangxinping@shzu.edu.cn (X.W.); 19915233520@163.com (B.C.); rmnmmtj@163.com (R.M.); liyu108@shzu.edu.cn (Y.L.); 2 Department of Hepatobiliary Surgery, The First Affiliated Hospital of Shihezi University School of Medicine, Shihezi 832000, China; yj17709937418@163.com (J.Y.); pxy13909937222@163.com (X.P.); 3 Department of NHC Key Laboratory of Prevention and Treatment of Central Asia High Incidence Diseases, The First Affiliated Hospital of Shihezi University School of Medicine, Shihezi 832000, China

**Keywords:** metabolic dysfunction-associated fatty liver disease, cardiovascular disease, Uyghur population, cohort study

## Abstract

In 2020, a group of international experts proposed a new term ‘metabolic dysfunction-associated fatty liver disease’ (MAFLD) to replace ‘non-alcoholic fatty liver disease’. This study aimed to describe the epidemic characteristics of MAFLD, incidence of cardiovascular disease (CVD), and relationship between MAFLD and incident CVD. In 2016, 12,794 Uyghur adults from Kashgar, Xinjiang, were grouped according to the presence or absence of MAFLD. The primary outcome was the occurrence of CVD events. Fatty liver was diagnosed using ultrasound. The prevalence of MAFLD was 16.55%. After excluding patients with previous CVD, 11,444 participants were followed up for a median period of 4.7 years. During the follow-up period, the overall CVD incidence was 10.40% (1190/11,444). The incidence of CVD in the patients with MAFLD was significantly higher than that in the non-MAFLD patients (18.38% vs. 9.02%, *p* < 0.001; multivariable-adjusted hazard ratio = 1.37, 95% CI = 1.20–1.56). The prevalence of MAFLD was relatively low, whereas the incidence of CVD was relatively high among the Uyghur adults in rural Xinjiang. Individuals with MAFLD have a higher risk of developing CVD independent of traditional cardiovascular risk factors, obesity, type 2 diabetes mellitus (T2DM), and dyslipidaemia.

## 1. Introduction

With modernisation and changes in lifestyles, the global burden of fatty liver disease related to metabolic disorders is increasing, affecting approximately 25.24% of the world’s adult population [1]. Currently, the prevalence of non-alcoholic fatty liver disease (NAFLD) in Chinese adults is approximately 29.2%, and it has become one of the main public health problems [2]. Hepatic lipotoxicity and mitochondrial dysfunction caused by NAFLD can lead to hepatocyte apoptosis, inflammation, and fibrosis [3]. NAFLD is a multisystem disease not only associated with liver disease, but also affecting many extrahepatic organ systems, including the cardiovascular system. An increasing number of studies have found that cardiovascular disease (CVD) is the leading cause of death among patients with NAFLD [4,5].

In 2020, international experts reached a consensus and declared that NAFLD be renamed as ‘metabolic-related fatty liver disease’ (MAFLD) [6]. Its definition comprises metabolic factors, including body mass index (BMI), blood glucose level, waist circumference (WC), blood pressure (BP), and blood lipid indicators. The diagnostic criteria for MAFLD, which do not overemphasize alcohol consumption and do not exclude other liver diseases, can be applied to patients in any clinical setting [6]. MAFLD diagnosis can better identify individuals with fatty liver and liver fibrosis with complicated metabolism because its definition is different from that of NAFLD [7,8,9,10]. Recent research has shown that MAFLD is associated with CVD risk and is considered an independent risk factor for CVD [11].

Xinjiang is located in the north-western region of China. The Uyghur community is one of the main ethnic groups in Xinjiang. Cold weather and inconvenient transportation in the area limit cultivation and transportation of fresh vegetables, respectively. Preliminary studies have found that Uyghur residents consume few vegetables but a high amount of staple food, red meat, dairy products, vegetable oil, and salt [12,13]. The prevalence of NAFLD is reportedly high among the Uyghurs in the urban areas [2]. Nevertheless, there is still a lack of epidemiological studies on the prevalence of MAFLD in the rural Uyghur populations. Furthermore, the association between MAFLD and CVD risk needs to be clarified in large-scale cohort studies.

This study aimed to investigate the prevalence of MAFLD and incidence of CVD in the Uyghur population in rural Xinjiang and explore the correlation between MAFLD and incident CVD.

## 2. Materials and Methods

### 2.1. Study Population

This cohort study was conducted in Kashgar, Xinjiang, China. Using typical sampling, the 51st Regiment of the Farm, the only regiment with Uyghurs as its main residents, was selected as the survey site. The target participants included all 14,321 Uyghur adults in the 6th, 8th, 11th, 12th, and 13th companies of the regiment. The baseline survey was conducted between August 2016 and September 2016. Three follow-ups were conducted in 2019, 2020, and 2021, until August 2021. The content of the follow-up was consistent with that of the baseline survey, and the outcome events were collected and assessed at each follow-up survey. Of the 14,321 Uyghur adults identified in that region, we excluded frequent floating populations, pregnant women, and individuals who were unable to participate in the survey process (*n* = 317). If the participant completed the baseline survey twice, we used the first data set. After excluding 971 participants with incomplete basic information and 239 participants without ultrasound information, the epidemic characteristics of MAFLD were analysed for the remaining 12,794 individuals. We further excluded 1064 participants who had a history of CVD, including ischaemic heart disease (*n* = 850) and stroke (*n* = 595). Thus, the longitudinal analysis comprised 11,444 individuals who completed the follow-up (follow-up rate: 97.6%) (Figure S1). All participants provided written informed consent. The Institutional Ethics Review Board (IERB) of the First Affiliated Hospital of Shihezi University School of Medicine approved this study (IERB no.: SHZ2010LL01).

### 2.2. Data Collection

Clinical examinations and questionnaire surveys were conducted by well-trained doctors, nurses, and investigators at the hospital’s physical examination centre. To ensure the accuracy of the outcomes, we also collected medical insurance and hospitalisation records for 2016–2019 and 2019–2021 in 2020 and 2021, respectively.

Clinical examination was conducted to record the height, weight, BMI, WC, systolic BP (SBP), diastolic BP (DBP), and presence of T2DM, hypertension, dyslipidaemia, or fatty liver. The final BP value was the average of three measurements. The Modification of Diet in Renal Disease study equation was used to calculate the estimated glomerular filtration rate (eGFR) [14]. T2DM [15] and hypertension [16] were diagnosed according to the established standards. Obesity (BMI ≥30 kg/m^2^) and overweight (BMI ≥25 kg/m^2^) [17] were defined based on the World Health Organization diagnostic criteria for the adult population. Abdominal obesity was defined as waist–height ratio (WHtR) more than or equal to 0.5 [18]. Dyslipidaemia [19] was defined as the presence of one or more of the following: total cholesterol (TC) ≥6.22 mmol/L, triglycerides (TG) ≥2.26 mmol/L, high-density lipoprotein cholesterol (HDL-C) <1.04 mmol/L, or low-density lipoprotein cholesterol (LDL-C) ≥4.14 mmol/L. Based on the above cut-offs, we separately defined high TC, high TG, low HDL-C, and high LDL-C, which were considered as other cardiometabolic risk factors [20]. Abdominal ultrasonography was performed by a professional clinician.

### 2.3. Biochemical Analysis

All participants fasted overnight before blood sample collection. Laboratory parameters recorded at baseline included TG, TC, HDL-C, LDL-C, fasting plasma glucose (FPG), alanine aminotransferase (ALT), aspartate aminotransferase (AST), gamma-glutamyl transferase (GGT), and serum creatinine (SCr) levels. All parameters were recorded using an Olympus AU 2700 automatic biochemical analyser (Olympus Diagnostics, Hamburg, Germany) at the Laboratory of the First Affiliated Hospital of Shihezi University School of Medicine. 

### 2.4. Questionnaire Survey

In the baseline and follow-up surveys, each participant underwent face-to-face interviews. We used standardized questionnaires to collect basic demographic information, lifestyle, disease history, family history, and medication status of the participants. Smoking was defined as an existing habit of smoking of over 100 cigarettes prior to the interview [21]. Drinking was defined as continuous alcohol consumption at least twice a month [22]. 

### 2.5. Key Definitions

MAFLD [6] was defined by the evidence of hepatic steatosis in addition to the presence of least one of the following three criteria: overweight/obesity (BMI ≥23.0 kg/m^2^ in Asian populations), T2DM, or metabolic dysregulation. Metabolic dysregulation was defined as the presence of at least two of the following metabolic abnormalities in those with lean/normal weight (BMI <23.0 kg/m^2^ in Asian populations): WC ≥90 cm and >80 cm in Asian men and women, respectively; BP ≥130/85 mmHg; TG ≥1.7 mmol/L; HDL-C <1.0 mmol/L and <1.3 mmol/L for men and women, respectively; and FPG in the range of 5.6–6.9 mmol/L. The homeostasis model assessment insulin resistance score and plasma high-sensitivity C-reactive protein level could not be evaluated in our study.

In the sensitivity analysis, we used another non-invasive method that differed from ultrasound and was based on the serum sample biomarkers to determine hepatic steatosis, i.e., fatty liver index (FLI ≥60) [23] and hepatic steatosis index (HSI >36) [24]. The FLI is recommended by international guidelines for use in large-scale epidemiological studies and has been verified in external populations such as Dutch whites, South Koreans, and the Taiwanese in China [25,26]. The detailed calculations are provided in Table S1.

### 2.6. Diagnosis of CVD

The primary outcome of this study was the first identified CVD diagnosis, defined as ischaemic heart disease (International Classification of Diseases 10th revision codes I20–25) or stroke (I60–64 and I69) during follow-up [27]. The CVD events were recorded using self-reported questionnaires and hospitalisation medical records. Self-reporting required an accompanying clinical diagnosis certificate. 

## 3. Statistical Analysis

Baseline characteristics are expressed as numbers with percentages or means with standard deviations. The Mann–Whitney U-test was used for the comparison of continuous variables between groups and the chi-square test for categorical variables. The Kaplan–Meier method was used to estimate the cumulative incidence of CVD events. Hazard ratios (HRs) and 95% confidence intervals (CIs) of CVD were calculated using Cox proportional risk models. The risk factors for CVD in the MAFLD group were determined through a single-factor analysis. The proportional hazard assumption was tested using Schoenfeld residuals. Following this, a forward stepwise approach was used to construct a multivariable model including variables that were significantly associated with the incidence of CVD in univariate analysis and the known traditional risk factors for CVD. A likelihood ratio test was used with a *p*-value < 0.05 as the threshold to test whether the added variables significantly improved the model. The final complete model determined by the step-forward method was adjusted for age, sex, SBP, DBP, HDL-C levels, eGFR, T2DM, and smoking. Statistical Product and Service Solutions version 26 (SPSS Inc., Chicago, IL, USA) and R version 4.1.1 (R Foundation for Statistical Computing, Vienna, Austria) were used for analysis.

We first conducted a sensitivity analysis using the FLI and HSI, a non-invasive method to evaluate hepatic steatosis, to describe the prevalence of MAFLD. We then performed a subgroup analysis to assess whether the correlation between MAFLD and CVD differed in the predetermined subgroups based on sex, age, and cardiometabolic risk factors.

## 4. Results

### 4.1. Baseline Characteristics

**Figure 1:**
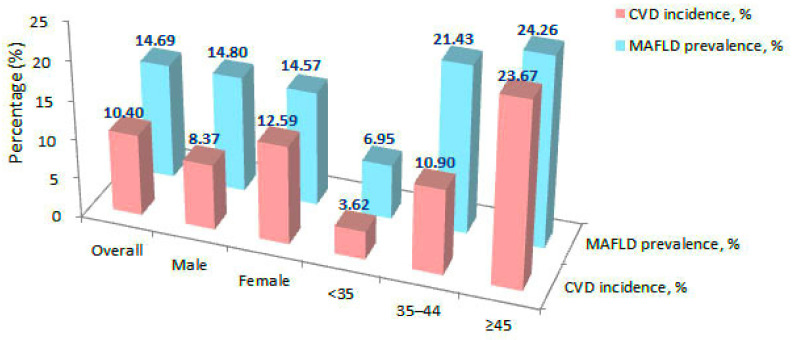
The prevalence of metabolic dysfunction-associated fatty liver disease and the incidence of cardiovascular disease according to sex and age in the overall study population (MAFLD, metabolic dysfunction-associated fatty liver; CVD, cardiovascular disease).

In the baseline data, the prevalence of MAFLD was 16.55% (2118/12,794). After excluding patients with prior CVD, 11,444 participants were finally included in the cohort study. Table 1 shows their baseline characteristics; the prevalence of MAFLD was 14.69% (1681/11,444). The cohort was followed up for a median period of 4.7 years, and the incidence of CVD reached 10.40% (1190/11,444) (Figure 1). The mean age and BMI of the participants was 36.47 ± 13.38 years and 25.77 ± 4.77 kg/m^2^, respectively. The proportion of the patients with MAFLD who were married, illiterate, or alcoholics was higher than that of the non-MAFLD patients. There were no statistical differences between the two groups in terms of sex, smoking, and family history of CVD. Among the patients with MAFLD, the prevalence of obesity, T2DM, and dyslipidaemia were 58.18%, 4.79%, and 52.83%, respectively. Individuals with obesity, T2DM, and dyslipidaemia were more likely to have MAFLD than those without (*p* < 0.05). Both MAFLD and non-MAFLD groups showed that overweight, obesity, high LDL-C, BMI, WC, SBP, DBP, HDL-C, SCr, and eGFR levels were associated with increased risk of CVD (all *p* < 0.05) (Table 1).

In addition, even in non-obesity, non-dyslipidaemia, and non-T2DM populations with relatively normal metabolic states, the MAFLD group was still significantly higher than the non-MAFLD group in terms of the parameter levels and CVD incidence (Table S2).

### 4.2. Incidence of CVD

**Figure 2:**
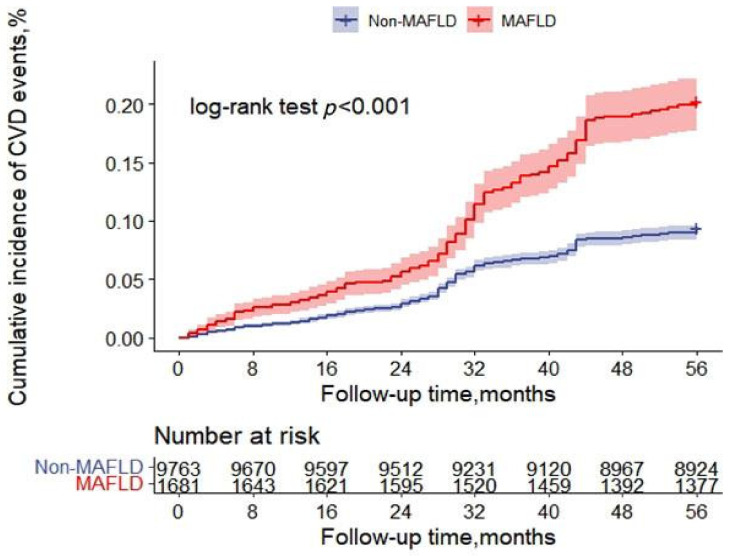
Kaplan–Meier estimates for cumulative cardiovascular disease incidence based on the presence of metabolic dysfunction-associated fatty liver disease (MAFLD, metabolic dysfunction-associated fatty liver; CVD, cardiovascular disease).

In total, 1190 (10.40%) new CVD events occurred during the follow-up period. The incidences of CVD in the MAFLD and non-MAFLD groups were 18.38% (309/1681) and 9.02% (881/9763), respectively. The Kaplan–Meier curve in Figure 2 shows that the cumulative incidence of CVD events was higher in patients with MAFLD than in those without (ꭓ^2^ = 132.79; *p* < 0.001; HR = 2.12, 95% CI = 1.86–2.42).

### 4.3. CVD Univariate and Multivariate Analysis

Univariate analysis revealed that factors such as sex, age, marital status, education, smoking, drinking, obesity, T2DM, dyslipidaemia, BMI, WC, SBP, DBP, GGT level, and eGFR were related to incident CVD (all *p* < 0.05). The model determined by the Cox stepwise-forward approach showed that sex, age, obesity, SBP, DBP, HDL-C, eGFR, and T2DM were significantly associated with an increased risk of CVD, independent of the other variables. Since smoking is a popular risk factor for CVD, we included it in the final model. Furthermore, as obesity is an important component of the MAFLD definition, we did not adjust for it. After multivariate adjustment, the incidence of CVD was still higher in the MAFLD group than in the non-MAFLD group (HR = 1.37, 95% CI = 1.20–1.56) (Table 2).

### 4.4. Sensitivity Analysis

A sensitivity analysis was performed as described below. First, the correlation between MAFLD and increased risk of CVD was found to be consistent in different biochemical steatosis models and ultrasound diagnoses (Table 2). Next, when the subgroup analysis was conducted by sex, age, abdominal obesity, overweight, low HDL-C, and high TG, MAFLD was still significantly associated with an increased risk of CVD (Table 3). Finally, the groupings based on the presence of MAFLD, obesity, T2DM, and dyslipidaemia showed that MAFLD with cardiometabolic risk factors had a higher CVD risk than other groups (Table 4 and Figure S2).

## 5. Discussion

This prospective cohort study from Xinjiang, China, is the first to report the prevalence of MAFLD (16.55%) in rural Uyghur adults. The risk of CVD was higher in the patients with MAFLD than in the non-MAFLD patients. However, as expected, the traditional CVD risk factors and metabolic factors played an important role in the relationship between MAFLD and CVD. Even after adjustment or subgroup analysis, the association between the two remained. This study revealed the independent effects of MAFLD on the incidence of CVD events.

In this study, the prevalence of MAFLD was lower than the average level reported in China. The prevalence of MAFLD varies greatly in different populations, depending on the race and geographic region [2]. In China, the prevalence of NAFLD ranges from 13% in rural areas to 43% in urban areas [28]. Nevertheless, in this study, we found that the prevalence of MAFLD based on the FLI score ≥60 was 22.72%, which was higher than that reported in the Japanese population [29]. This may be related to overweight, WC, and TG levels in the Uyghur population in this study. The Uyghurs have a mix of genes of white and East Asian populations [30,31]. Research has revealed that Asians store less subcutaneous fat. Their visceral fat content is significantly higher than that of whites [32]. Notably, visceral fat reflects metabolic abnormalities better than subcutaneous fat does [33]. Recently, Wang et al. [12] found that the proportion of obese individuals with normal metabolism in the Uyghur community was higher than that of individuals from the Han community. Therefore, when the Uyghurs gain weight, the fat distribution that is conducive to metabolic health may be one of the reasons why the prevalence of MAFLD in this study was lower than the Chinese average level. It may also be related to the following reasons: the average age of the population in this study was relatively low; influenced by the religious culture of smoking ban and prohibition of alcohol consumption, the population had a low smoking and drinking rate; the occupation was mainly manual labour; low-income farmers were the main residents; the intake of refined food was small; and the prevalence of T2DM was low.

Although the definition of MAFLD was proposed in 2020, there are limited studies on the relationship between MAFLD and CVD. Many studies have shown that NAFLD is independently associated with an increased risk of CVD [34,35,36]; however, some studies showed different results [37,38]. MAFLD comprises an improved and extended version of NAFLD. As compared to NAFLD, MAFLD reflects metabolic dysfunction more accurately, and some scholars believe that it has a closer relationship with CVD [7]. Moreover, the association between MAFLD and CVD fulfilled biological rationality, and both are manifestations of end-organ damage resulting from the metabolic syndrome. The existence of insulin resistance (IR) is believed to play a central role in the link between the two [39]. Recently, studies have shown a bidirectional correlation between MAFLD and IR. An observational study confirmed the hypothesis that the presence of IR increased the risk of advanced fibrosis, which led to worsening of the liver status [40]. Insulin resistance promotes increased hepatic lipogenesis and excess accumulation of free fatty acids (FFA) in the liver. At the same time, the accumulation of FFA also induces changes in the insulin signalling pathway by activating serine kinases, which in turn aggravates the IR state [41]. Several experimental studies found that lowering IR levels by improving the liver status through direct-acting antiviral therapy reduced the CVD risk and T2DM incidence [42,43]. The interaction between MAFLD and IR increases the levels of VLDL particles and TG, resulting in insulin receptor dysfunction, thereby mobilizing liver adipose tissue to transport to peripheral tissues and increasing the risk of CVD [44]. In addition to IR, complex underlying mechanisms between MAFLD and CVD include endothelial dysfunction and activation of inflammatory pathways [45]. The pro-inflammatory state and increased oxidative stress in patients with MAFLD can lead to endothelial dysfunction and induce vascular inflammation, thereby promoting the formation of atherosclerotic plaque. All of the above mechanisms can lead to changes in cardiac structure and diastolic dysfunction [46].

This study found that the incidence of CVD among the Uyghurs in rural Xinjiang was high, and the incidence in the MAFLD group was significantly higher than that in the non-MAFLD group. The HR value between the two groups suggests that the association between MAFLD and CVD incidence was moderately close. The incidence of CVD in this study was higher than the 7.84% reported by Ji et al. in the Han Chinese population [47]. This could be attributed to the Xinjiang Uyghurs’ unique diet, lifestyle, the natural environment of the region, and genetic factors. Uyghurs are an ethnic group with the lowest rate of interethnic marriages in China [48]. Their cultural beliefs and living customs are weakly influenced by mainstream Han culture, and their population flow is small; hence, they retain their unique ethnic genes. They are considered a high-risk group for hypertension, metabolic syndrome, obesity, and coronary heart disease [49,50,51,52], which may lead to a higher incidence of CVD.

In this study, we found that after adjustment of the complete model, MAFLD was still associated with an increased risk of CVD. Recently, a study by Lee et al. [7] on middle-aged Koreans aged 40–64 years showed that this correlation remained significant after multivariate adjustment (HR = 1.52, 95% CI = 1.51–1.54). Another Japanese retrospective cohort study showed that the risk of CVD in patients with MAFLD increased by 1.89 times (95% CI = 1.78–2.01) [29]. Recently, a cohort study of 55–70-year-old individuals from Shanghai, China, also showed similar results; however, it was not observed in the subgroup of patients with excessive alcohol consumption and viral hepatitis [11]. Similarly, we did not observe a correlation between MAFLD and CVD among those who consumed alcohol. This could be attributed to the low rate of alcohol consumption in this population [53]. We also noticed that the HR in our study was lower than that observed in the previous studies. This could be owing to the age composition of the study population and differences in genetic backgrounds. These previous studies mostly involved middle-aged and older individuals aged >40 years, whose metabolic capacity is relatively poor and for whom the risk of CVD and related diseases is high. The population in our study was relatively young, and the association between MAFLD and CVD was observed in the two subgroups aged ≥35 and <35 years, which is more convincing. With respect to genetic factors, the previous studies were conducted in East Asian populations, whose visceral fat content is higher than that of the Uyghurs. The IR is stronger in visceral fat than in subcutaneous fat [54,55], and IR may be one of the most important factors connecting visceral fat and CVD [56]. We also found that the incidence of CVD was more strongly related to MAFLD in men than in women. This can be explained by sex-related differences in fat distribution. Since men are more likely to accumulate visceral fat than women [57], they are at a higher risk of CVD.

Our findings showed that the relationship between MAFLD and CVD was independent of the cardiometabolic risk factors such as abdominal obesity, overweight, high TG, low HDL-C, obesity, T2DM, and dyslipidaemia. The prevalence of these diseases in the patients with MAFLD was significantly higher than that in the non-MAFLD patients. This suggests that patients with MAFLD are prone to clustered cardiometabolic risk factors, which may increase the risk of CVD. To observe the relationship between MAFLD and CVD in different metabolic states, we conducted a subgroup analysis and found that the MAFLD with cardiometabolic risk factors group had the highest risk of CVD when compared with other groups. Even in the non-obesity, non-T2DM, non-dyslipidaemia normal metabolic groups, MAFLD still increased the risk of CVD. That is, regardless of the presence of cardiometabolic risk factors in patients with MAFLD, their risk of CVD was higher than that of the non-MAFLD group. Therefore, the focus should not just be on the metabolically abnormal population, but the MAFLD status of more populations should be monitored to prevent CVD events.

## 6. Strengths and Limitations

The main strength of this study is the long-term follow-up of a large number of participants. In addition to ultrasound, we also used the biochemical steatosis model with the FLI and HSI to diagnose MAFLD, adding to the robustness of the results. Nevertheless, our study had some limitations. Firstly, as only the Uyghur population was studied, the results cannot be generalised to other Asian or non-Asian ethnic groups or populations. Second, the population studied had a mean age of 36.47 years. Thus, the results cannot be generalised to an older population. In addition, the diagnosis of MAFLD only at baseline cannot assess the impact of dynamic MAFLD changes on the incidence of CVD. Further research should be conducted on the basis of a suitable MAFLD dynamic evaluation system, and we did not detect the prevalence of other liver diseases. Social security, hospitalisation, and surgical records alone are insufficient to assess the effect of MAFLD combined with other liver diseases on the incidence of CVD. Furthermore, data on relevant medications were lacking. Most of the participants had poor health awareness; they did not remember medication use or did not receive treatment. Finally, MAFLD gradually progresses from hepatic steatosis to fibrosis, hepatitis, cirrhosis, and other end-stage liver diseases. Further research is warranted to evaluate the effect of the existence and severity of liver fibrosis on the risk of CVD.

In conclusion, MAFLD is associated with increased CVD risk. Patients with MAFLD should be monitored and interventions should be timely to better manage the CVD high-risk groups through stratification and make precision medicine possible.

## Figures and Tables

**Table 1:** Baseline characteristics of the participants grouped by the presence of metabolic dysfunction-associated fatty liver disease and new cardiovascular disease events.

Variable	Overall	MAFLD				Non-MAFLD			
		*n*_1_	CVD	Non-CVD	*p*_1_	*n*_2_	CVD	Non-CVD	*p*_2_
*n*	11,444 (100)	1681 (14.69)	308 (18.32)	1373 (81.68)		9763 (85.31)	882 (9.03)	8881 (90.96)	
Age (years) ^a^	36.47 ± 13.38	42.85 ± 11.57	48.96 ± 11.16	41.47 ± 11.20	<0.001	35.38 ± 13.36	47.25 ± 14.06	34.20 ± 12.70	<0.001
Sex					<0.001				<0.001
Male	5940 (51.90)	879 (52.29)	124 (40.26)	755 (54.99)		5061 (51.84)	373 (42.29)	4688 (52.79)	
Female	5504 (48.10)	802 (47.71)	184 (59.74)	618 (45.01)		4702 (48.16)	509 (57.71)	4193 (47.21)	
Marital status ^a^					0.296				<0.001
No	2148 (18.77)	154 (9.16)	33 (10.71)	121 (8.81)		1994 (20.42)	139 (15.76)	1855 (20.89)	
Yes	9296 (81.23)	1527 (90.84)	275 (89.29)	1252 (91.19)		7769 (79.58)	743 (84.24)	7026 (79.11)	
Education ^a^					<0.001				<0.001
Illiteracy	4334 (37.87)	754 (44.85)	171 (55.52)	583 (42.46)		3580 (36.67)	496 (56.24)	3084 (34.73)	
Primary school	3042 (26.58)	458 (27.25)	68 (22.08)	390 (28.40)		2584 (26.47)	208 (23.58)	2376 (26.75)	
≥Junior high school	4068 (35.55)	469 (27.90)	69 (22.40)	400 (29.13)		3599 (36.86)	178 (20.18)	3421 (38.52)	
Smoking					<0.001				<0.001
No	9403(82.17)	1383(82.27)	279 (90.58)	1104 (80.41)		8020(82.15)	770 (87.30)	7250 (81.63)	
Yes	2041(17.83)	298(17.73)	29 (9.42)	269 (19.59)		1743(17.85)	112 (12.70)	1631 (18.37)	
Drinking ^b^					0.001				0.372
No	10,841 (94.73)	1571 (93.46)	301 (97.73)	1270 (92.50)		9270 (94.95)	843 (95.58)	8427 (94.89)	
Yes	603 (5.27)	110 (6.54)	7 (2.27)	103 (7.50)		493 (5.05)	39 (4.42)	454 (5.11)	
Overweight ^a^	5958 (52.06)	1572 (93.52)	297 (96.43)	1275 (92.86)	0.022	4386 (44.92)	556 (63.04)	3830 (43.13)	<0.001
Abdominal obesity ^a^	8394 (73.35)	1605 (63.36)	295 (95.78)	1310 (95.41)	0.779	6789 (69.54)	743 (84.24)	6046 (68.08)	<0.001
Obesity ^a^					<0.001				<0.001
No	9421 (82.32)	703 (41.82)	101 (32.79)	602 (43.85)		8718 (89.30)	703 (79.71)	8015 (90.25)	
Obese class Ⅰ	1574 (13.75)	274 (16.30)	124 (40.26)	150 (10.92)		1300 (13.32)	543 (61.56)	757 (8.52)	
Obese class Ⅱ	367 (3.21)	98 (5.83)	71 (23.05)	27 (1.97)		269 (2.76)	182 (20.63)	87 (0.98)	
Obese class Ⅲ	82 (0.72)	14 (0.83)	12 (3.90)	2 (0.15)		68 (0.70)	46 (5.22)	22 (0.25)	
T2DM ^a^					0.055				<0.001
No	10,896 (95.21)	1498 (89.11)	265 (86.04)	1233 (89.80)		9398 (96.26)	812 (92.06)	8586 (96.68)	
Yes	548 (4.79)	183 (10.89)	43 (13.96)	140 (10.20)		365 (3.74)	70 (7.94)	295 (3.32)	
FPG level ^a^					0.278				0.012
≤6.0	10,442 (91.24)	1386 (82.45)	248 (80.52)	1138 (82.88)		9056 (92.76)	789 (89.46)	8267 (93.09)	
6.1–6.9	495 (4.33)	53 (3.15)	19 (6.17)	34 (2.48)		442 (4.53)	107 (12.13)	335 (3.77)	
≥7.0	507 (4.43)	100 (5.95)	41 (13.31)	59 (4.30)		407 (4.17)	128 (14.51)	279 (3.14)	
Dyslipidaemia ^a^					0.157				<0.001
No	8099 (70.77)	798 (47.47)	135 (43.83)	663 (48.29)		7301 (74.78)	580 (65.76)	6721 (75.68)	
Yes	3345 (29.23)	883 (52.53)	173 (56.17)	710 (51.71)		2462 (25.22)	302 (34.24)	2160 (24.32)	
High LDL ^a^	276 (2.41)	59 (3.51)	24 (7.79)	35 (2.55)	0.009	217 (2.22)	58 (6.58)	159 (1.79)	<0.001
Low HDL ^a^	976 (8.53)	187 (11.12)	69 (22.40)	118 (8.59)	1.000	789 (8.08)	164 (18.59)	625 (7.04)	<0.001
High TG ^a^	2124 (18.56)	272 (16.18)	119 (38.64)	153 (11.14)	1.000	1852 (18.97)	541 (61.34)	1311 (14.76)	0.074
High TC ^a^	874 (7.64)	105 (6.25)	38 (12.34)	67 (4.88)	0.068	769 (7.88)	227 (25.74)	542 (6.10)	0.080
Family history of CVD	1416 (12.37)	222 (13.21)	23 (7.47)	199 (14.49)	0.001	1194 (12.23)	113 (12.81)	1081 (12.17)	0.58
Family history of T2DM ^a^	435 (3.80)	101 (6.01)	13 (4.22)	88 (6.41)	0.144	334 (3.42)	38 (4.31)	296 (3.33)	0.129
BMI (kg/m^2^) ^a^	25.78 ± 4.78	31.19 ± 4.94	32.13 ± 4.34	30.98 ± 5.04	<0.001	24.85 ± 4.08	26.58 ± 4.16	24.68 ± 4.03	<0.001
WC (cm) ^a^	90.01 ± 13.16	101.91 ± 12.31	104.05 ± 12.66	101.44 ± 12.18	<0.001	87.96 ± 12.17	92.16 ± 12.80	87.54 ± 12.03	<0.001
SBP (mm Hg) ^a^	126.59 ± 17.96	133.94 ± 19.30	142.14 ± 21.52	132.10 ± 18.28	<0.001	125.32 ± 17.41	136.66 ± 22.71	124.19 ± 16.37	<0.001
DBP (mm Hg) ^a^	74.15 ± 11.96	78.59 ± 12.63	82.06 ± 13.12	77.81 ± 12.39	<0.001	73.38 ± 11.67	78.14 ± 13.03	72.91 ± 11.42	<0.001
FPG (mmol/L) ^a^	4.92 ± 1.84	5.48 ± 2.77	5.66 ± 3.14	5.44 ± 2.68	0.726	4.83 ± 1.61	5.05 ± 2.12	4.81 ± 1.54	0.428
TG (mmol/L) ^a^	1.69 ± 1.44	2.39 ± 1.87	2.33 ± 1.55	2.41 ± 1.94	0.904	1.57 ± 1.32	1.72 ± 1.22	1.56 ± 1.33	<0.001
TC (mmol/L) ^a^	4.72 ± 2.04	5.24 ± 1.99	5.02 ± 1.08	5.30 ± 2.14	0.087	4.63 ± 2.03	4.74 ± 1.40	4.62 ± 2.08	<0.001
HDL-C (mmol/L) ^a^	1.57 ± 0.56	1.52 ± 0.59	1.41 ± 0.63	1.55 ± 0.57	<0.001	1.58 ± 0.78	1.45 ± 0.55	1.59 ± 0.56	<0.001
LDL-C (mmol/L) ^a^	2.63 ± 0.83	2.84 ± 1.06	3.00 ± 1.83	2.81 ± 0.79	0.106	2.59 ± 0.78	2.69 ± 0.80	2.58 ± 0.78	<0.001
ALT (IU/L) ^a^	24.59 ± 13.69	26.00 ± 12.98	26.61 ± 14.17	25.87 ± 12.70	0.45	24.35 ± 13.80	23.80 ± 10.29	24.40 ± 14.10	0.033
AST (IU/L) ^a^	30.47 ± 24.69	38.33 ± 28.62	36.34 ± 31.33	38.78 ± 27.97	0.001	29.12 ± 23.70	28.82 ± 23.96	29.15 ± 23.67	0.194
GGT (IU/L) ^a^	19.15 ± 16.52	25.81 ± 19.93	23.74 ± 14.60	23.79 ± 17.37	0.347	17.62 ± 15.23	19.68 ± 15.23	18.22 ± 10.50	0.257
SCr (mol/L) ^a^	71.60 ± 16.17	72.91 ± 16.97	68.94 ± 16.76	73.80 ± 16.90	<0.001	71.38 ± 16.02	69.52 ± 17.45	71.56 ± 15.86	<0.001
eGFR (ml/min/1.73 m^2^) ^a^	108.39 ± 36.93	103.79 ± 38.49	88.39 ± 35.91	107.25 ± 38.21	<0.001	109.17 ± 36.60	91.58 ± 39.09	110.92 ± 35.87	<0.001
CVD incidence ^a^	1190 (10.40)	308 (18.32)				882 (9.03)			
Follow-up, years	4.44 ± 0.78	4.26 ± 1.00				4.47 ± 0.74			

Values are presented as means ± standard deviation or *n* (%). ^a^ *p* < 0.001 for *n*_1_ compared to *n*_2_; ^b^ *p* < 0.05 for *n*_1_ compared to *n*_2_. *p*_1_ = the results of the chi-square or Mann–Whitney U-test for differences in baseline parameters of the participants with MAFLD between the CVD group and the non-CVD group; *p*_2_ = the results of the chi-square or Mann–Whitney U-test for differences in baseline parameters of the participants with non-MAFLD between the CVD group and the non-CVD group.

**Table 2:** Cox regression model of the relationship between cardiovascular disease and metabolic dysfunction-associated fatty liver disease diagnosed using different steatosis models.

Group	*n* (%)	CVD Events	Rate *	HR (95% CI)		
				Model 1	Model 2	Model 3
US						
Non-MAFLD	9763 (85.31)	882	549.5	Reference	Reference	Reference
MAFLD	1681 (14.69)	308	1160.2	2.12 (1.86–2.42)	1.54 (1.36–1.76)	1.36 (1.19–1.55)
FLI						
Non-MAFLD	8843 (77.27)	718	438.7	Reference	Reference	Reference
MAFLD	2601 (22.72)	472	1023.4	2.36 (2.10–2.65)	1.66 (1.48–1.87)	1.37 (1.21–1.55)
HSI						
Non-MAFLD	7626 (66.64)	557	397.3	Reference	Reference	Reference
MAFLD	3818 (33.36)	633	920.9	2.39 (2.13–2.67)	1.54 (1.37–1.74)	1.30 (1.15–1.46)

Model 1 was unadjusted. Model 2 was adjusted for age and sex. Model 3 was further adjusted for SBP, DBP, HDL-C, eGFR, T2DM, and smoking. * Rate per 10,000 person-years.

**Table 3:** Subgroup analysis of the relationship between metabolic dysfunction-associated fatty liver disease and cardiovascular disease.

Subgroup	Non-MAFLD		MAFLD		HR (95% CI)
	CVD Events	Rate *	CVD Events	Rate *	
Sex					
male	373	413.8	124	796.9	1.35 (1.10–1.66)
female	509	569.1	184	1330.2	1.33 (1.12–1.58)
Age					
<35	183	190.4	29	445.4	1.77 (1.18–2.65)
≥35	699	889.4	279	1253.4	1.21 (1.05–1.39)
Smoking	112	387.7	29	561.2	1.11(0.73,1.68)
Drinking	39	474.3	7	348.2	0.45 (0.19–1.03)
Overweight	556	684.6	297	1088.6	1.27 (1.11–1.47)
Abdominal obesity	743	590.1	295	1058.6	1.32 (1.15–1.51)
High LDL	35	1052.7	24	1885.1	1.58 (0.92–2.70)
Low HDL	118	923.6	69	1593.5	1.90 (1.40–2.57)
High TG	153	579.5	119	940.7	1.55 (1.22–1.98)
High TC	67	599.5	38	806.4	1.08 (0.73–1.62)

All models were adjusted for sex, age, SBP, DBP, HDL-C, eGFR, T2DM, and smoking. * Rate per 10,000 person-years.

**Table 4:** Subgroup analysis according to the presence of metabolic dysfunction-associated fatty liver disease and/or obesity, dyslipidaemia, T2DM.

Subgroup		CVD Events	Rate *	HR (95% CI)		
				Model 1	Model 2	Model 3
MAFLD	Obesity					
−	−	703	490.9	Reference	Reference	Reference
−	+	179	842.7	2.21 (1.88–2.61)	1.60 (1.36–1.89)	1.31 (1.11–1.55)
+	−	101	1059.3	1.84 (1.49–2.27)	1.37 (1.12–1.69)	1.30 (1.05–1.60)
+	+	207	1393.2	2.75 (2.35–3.21)	1.86 (1.59–2.17)	1.52 (1.30–1.79)
MAFLD	Dyslipidaemia					
−	−	580	422.1	Reference	Reference	Reference
−	+	302	686.4	1.58 (1.37–1.81)	1.19 (1.03–1.37)	1.12 (0.97–1.29)
+	−	135	1022.6	2.20 (1.83–2.66)	1.46 (1.21–1.76)	1.28 (1.06–1.55)
+	+	173	1065.5	2.63 (2.22–3.12)	1.81 (1.53–2.15)	1.56 (1.31–1.86)
MAFLD	T2DM					
−	−	812	465.4	Reference	Reference	Reference
−	+	70	1095.9	2.35 (1.84–3.00)	1.40 (1.09–1.79)	1.34 (1.05–1.71)
+	−	265	1013.5	2.14 (1.86–2.46)	1.55 (1.35–1.78)	1.38 (1.20– 1.59)
+	+	43	1311.6	2.96 (2.18–4.02)	1.82 (1.34–2.48)	1.64 (1.20–2.23)

Model 1 was unadjusted. Model 2 was adjusted for age and sex. Model 3 was further adjusted for SBP, DBP, HDL-C, eGFR, T2DM, and smoking. * Rate per 10,000 person-years.

## Data Availability

Some or all datasets generated during and/or analysed during the current study are not publicly available but are available from the corresponding author on reasonable request.

## Supplementary Materials

The following are available online at https://www.mdpi.com/article/10.3390/nu14122361/s1, Figure S1: Flow chart of inclusion and exclusion criteria of the study participants., Figure S2: Kaplan–Meier estimates for cumulative cardiovascular disease incidence based on the presence of metabolic dysfunction-associated fatty liver disease, obesity, type 2 diabetes mellitus, dyslipidaemia; Table S1: Detailed calculation of non-invasive evaluation indicators of fatty liver index and hepatic steatosis index, Table S2: Baseline characteristics of non-obesity, non-dyslipidaemia, non-T2DM populations.

## Abbreviations

MAFLD: metabolic dysfunction-associated fatty liver disease; NAFLD, non-alcoholic fatty liver disease; CVD, cardiovascular disease; BMI, body mass index; WC, waist circumference; SBP, systolic blood pressure; DBP, diastolic blood pressure; FPG, fasting plasma glucose; HDL-C, high-density lipoprotein cholesterol; LDL-C, low-density lipoprotein cholesterol; TG, triglyceride; TC, total cholesterol; ALT, alanine aminotransferase; AST, aspartate aminotransferase; GGT, gamma-glutamyl transferase; SCr, serum creatinine; US, ultrasound; FLI, fatty liver index; HR, hazard ratio; T2DM, type 2 diabetes mellitus; FPG, fasting plasma glucose; eGFR, estimated glomerular filtration rate; WHtR, waist height ratio.
