# Supplementary material for: Metabolic Dysfunction-Associated Fatty Liver Disease Is Associated with the Risk of Incident Cardiovascular Disease: A Prospective Cohort Study in Xinjiang
Source: Nutrients. 2022 Jun 7;14(12):2361. doi: 10.3390/nu14122361 (PMC9231197; doi:10.3390/nu14122361)
Supplement: Supplementary file 1 [file nutrients-14-02361-s001.zip › nutrients-1701716-supplementary.pdf]

Supplementary Table S1. Detailed calculation of non-invasive evaluation indicators of fatty liver index and hepatic steatosis index

Definition	Criteria
Hepatic steatosis	FLI ≥60
	FLI= $\frac{e^{0.953\times \log_e(TG)+0.139\times BMI+0.718\times \log_e(GGT)+0.053\times WC-15.745}}{1+e^{0.953\times \log_e(TG)+0.139\times BMI+0.718\times \log_e(GGT)+0.053\times WC-15.745}}\times 100$
	HSI >36
	HSI=8 × (ALT/AST ratio) + BMI (+2, if female; +2, if diabetes mellitus)

Supplementary Table S2. Baseline characteristics of non-obesity, non-dyslipidaemia, non-T2DM populations

Variable	Non Obesity			Non Dyslipidaemia			Non T2DM		
	MAFLD	Non MAFLD	P_1	MAFLD	Non MAFLD	P_2	MAFLD	Non MAFLD	P_3
n	703 (7.46)	8718 (92.54)		798 (9.85)	7301 (90.15)		1,498 (13.75)	9398 (86.25)	
Age (years)	42.15 ± 11.87	34.67 ± 13.30	<0.001	42.00 ± 11.49	33.77 ± 13.09	<0.001	42.31 ± 11.56	35.02 ± 13.21	<0.001
Sex			0.001			<0.001			0.967
Male	419 (59.60)	4612 (52.90)		339 (42.48)	3589 (49.16)		776 (51.80)	4863 (51.75)	
Female	284 (40.40)	4106 (47.10)		459 (57.52)	3712 (50.84)		722 (48.20)	4535 (48.25)	
Marital status			<0.001			<0.001			<0.001
No	73 (10.38)	1890 (21.68)		85 (10.65)	1664 (22.79)		139 (9.28)	1941 (20.65)	
Yes	630 (89.62)	6828 (78.32)		713 (89.35)	5637 (77.21)		1359 (90.72)	7457 (79.35)	
Education			<0.001			<0.001			<0.001
Illiteracy	295 (41.96)	3068 (35.19)		365 (45.74)	2518 (34.49)		668 (44.59)	3393 (36.10)	
Primary school	191 (27.17)	2277 (26.12)		207 (25.94)	1877 (25.71)		409 (27.30)	2478 (26.37)	
⩾Junior high school	217 (30.87)	3373 (38.69)		226 (28.32)	2906 (39.80)		421 (28.10)	3527 (37.53)	
Smoking			0.02			0.066			0.848
No	548 (77.95)	7107 (81.52)		689 (86.34)	6121 (83.84)		1231 (82.18)	7742 (82.38)	
Yes	155 (22.05)	1611 (18.48)		109 (13.66)	1180 (16.16)		267 (17.82)	1656 (17.62)	
Drinking			0.006			0.011			0.011
No	650 (92.46)	8271 (94.87)		757 (94.86)	6978 (95.58)		1400 (93.46)	8931 (95.03)	
Yes	53 (7.54)	447 (5.13)		41 (5.14)	323 (4.42)		98 (6.54)	467 (4.97)	
Obesity						<0.001			<0.001
No	–	–		354 (44.36)	6672 (91.38)		631 (42.12)	8434 (89.74)	
Yes	–	–		444 (55.64)	629 (8.62)		867 (57.88)	964 (10.26)	
T2DM			<0.001			<0.001			
No	631 (89.76)	8434 (96.74)		745 (93.36)	7121 (97.53)		–	–	
Yes	72 (10.24)	284 (3.26)		53 (6.64)	180 (2.47)		–	–	
Dyslipidaemia			<0.001						<0.001
No	354 (50.36)	6672 (76.53)		–	–		745 (49.73)	7121 (75.77)	
Yes	349 (49.64)	2046 (23.47)		–	–		753 (50.27)	2277 (24.23)	
Family history of CVD	102 (14.51)	1058 (12.14)	0.065	98 (12.28)	871 (11.93)	0.772	198 (13.22)	1154 (12.28)	0.306
Family history of T2DM	46 (6.54)	282 (3.23)	<0.001	42 (5.26)	243 (3.33)	0.005	85 (5.67)	310 (3.30)	<0.001
BMI (kg/m^2)	27.07 ± 2.15	23.92 ± 3.09	<0.001	30.80 ± 4.30	24.36 ± 4.01	<0.001	31.16 ± 4.95	24.78 ± 4.05	<0.001

WC (cm)	95.96 ± 10.95	86.37 ± 11.32	<0.001	100.07 ± 11.64	86.56 ± 11.76	<0.001	101.46 ± 12.20	87.72 ± 12.10	<0.001
SBP (mm Hg)	131.33 ± 17.16	124.29 ± 16.78	<0.001	133.29 ± 19.02	123.93 ± 16.61	<0.001	133.36 ± 19.03	125.02 ± 17.23	<0.001
DBP (mm Hg)	77.23 ± 11.46	72.80 ± 11.40	<0.001	78.12 ± 13.43	72.80 ± 11.42	<0.001	78.47 ± 12.71	73.25 ± 11.64	<0.001
FPG (mmol/L)	5.38 ± 2.80	4.79 ± 1.46	<0.001	5.00 ± 1.68	4.72 ± 1.32	<0.001	4.85 ± 0.91	4.64 ± 0.83	<0.001
TG (mmol/L)	2.38 ± 1.99	1.52 ± 1.26	<0.001	1.37 ± 0.47	1.18 ± 0.49	<0.001	2.25 ± 1.76	1.54 ± 1.25	<0.001
TC (mmol/L)	5.23 ± 1.93	4.59 ± 1.98	<0.001	4.68 ± 0.83	4.34 ± 0.89	<0.001	5.22 ± 2.07	4.60 ± 2.04	<0.001
HDL (mmol/L)	1.53 ± 0.57	1.59 ± 0.55	<0.001	1.60 ± 0.54	1.62 ± 0.49	0.002	1.52 ± 0.58	1.58 ± 0.55	<0.001
LDL (mmol/L)	2.78 ± 0.81	2.58 ± 0.77	<0.001	2.66 ± 0.65	2.51 ± 0.64	<0.001	2.84 ± 1.07	2.59 ± 0.78	<0.001
ALT (IU/L)	25.86 ± 13.19	24.22 ± 13.50	<0.001	25.14 ± 11.95	24.06 ± 14.04	<0.001	25.93 ± 13.02	24.29 ± 13.84	<0.001
AST (IU/L)	37.43 ± 24.49	28.71 ± 23.17	<0.001	35.33 ± 29.81	27.52 ± 20.95	<0.001	38.24 ± 29.10	28.86 ± 23.50	<0.001
GGT (IU/L)	23.43 ± 16.83	18.07 ± 10.21	<0.001	20.66 ± 13.32	17.55 ± 8.85	<0.001	23.13 ± 16.15	18.22 ± 10.67	<0.001
SCr (μmol/L)	74.17 ± 17.91	71.35 ± 15.94	<0.001	70.70 ± 15.03	70.81 ± 14.15	0.817	72.84 ± 16.99	71.25 ± 15.24	<0.001
eGFR (ml/min/1.73 m^2)	107.24 ± 40.31	109.95 ± 36.44	0.027	100.12 ± 34.21	109.84 ± 32.99	<0.001	104.25 ± 38.50	109.33 ± 35.05	<0.001
CVD incidence	101 (14.37)	703 (8.06)	<0.001	135 (16.92)	580 (7.94)	<0.001	265 (17.69)	812 (8.64)	<0.001
Follow-up, years	4.32 ± 0.96	4.49 ± 0.70	<0.001	4.32±0.92	4.49 ± 0.70	<0.001	4.28 ± 0.98	4.47 ± 0.72	<0.001

Values are presented as means ± standard or n (%).

P_1, P_2, and P_3 mean the differences in baseline parameters between the MAFLD group and the non-MAFLD group in the non-obesity, non-dyslipidaemia, and non-T2DM populations, respectively.

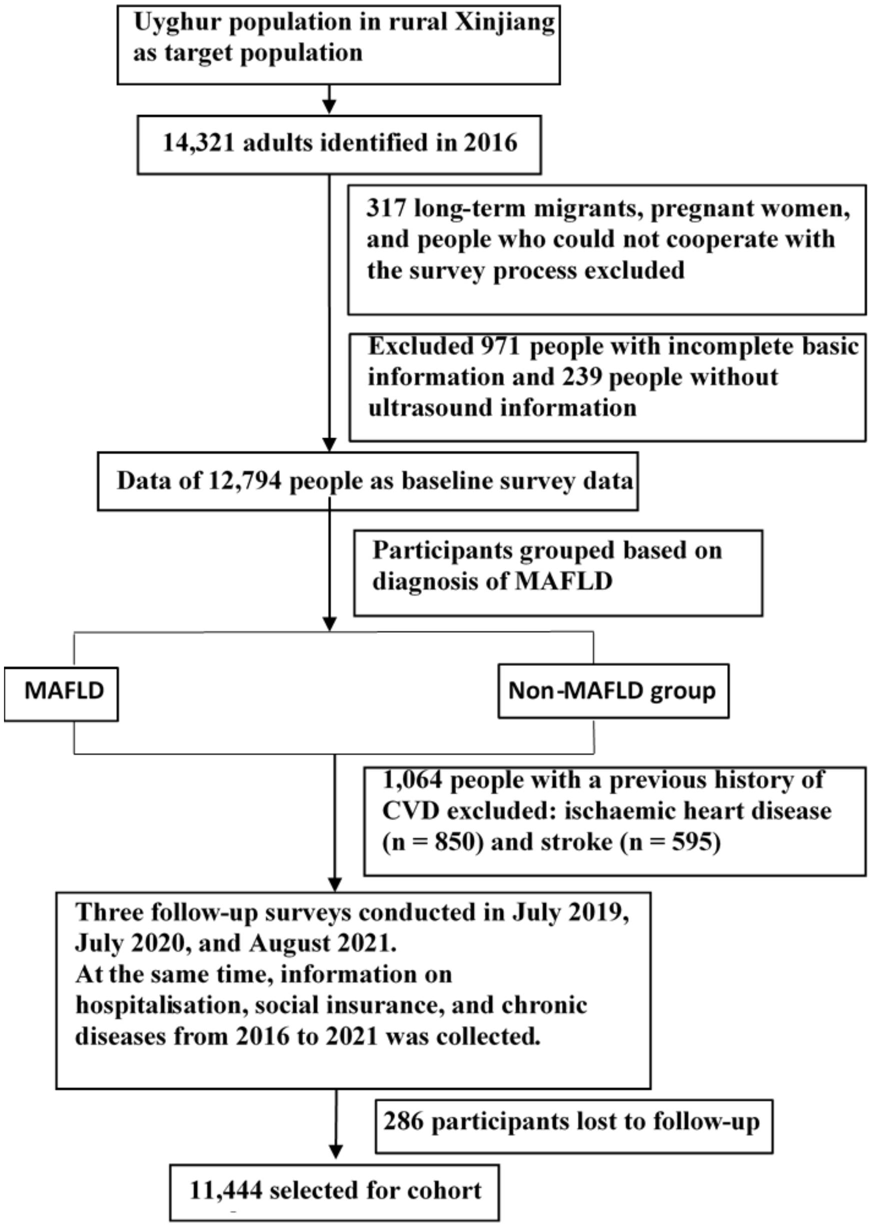

Supplementary Figure S1. Flow chart of inclusion and exclusion criteria of the study participants.

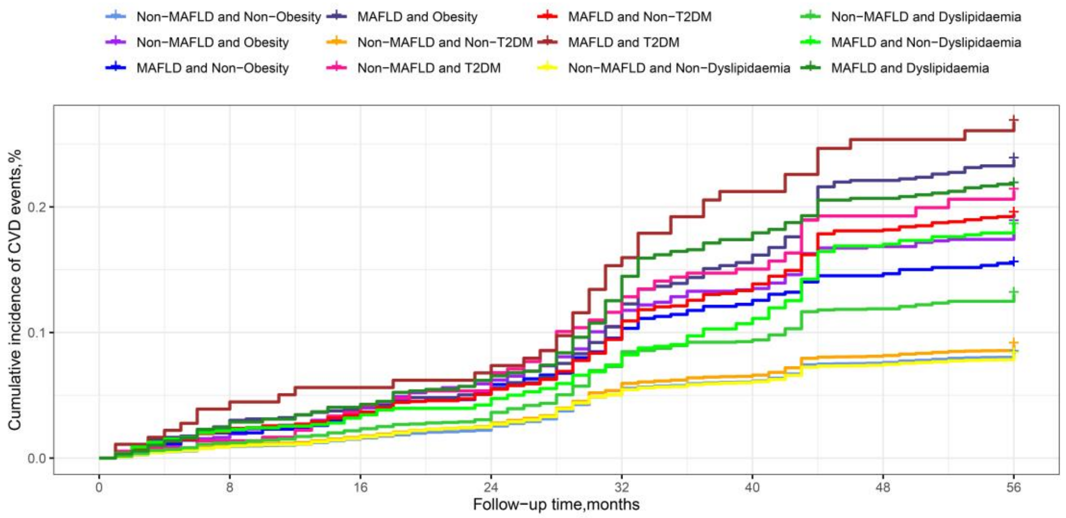

	Wald χ^2	log-rank test P value
MAFLD, Obesity	223.754	< 0.001
MAFLD, T2DM	174.891	< 0.001
MAFLD, Dyslipidaemia	166.464	< 0.001

Supplementary Figure S2. Kaplan–Meier estimates for cumulative cardiovascular disease incidence based on the presence of metabolic dysfunction-associated fatty liver disease, obesity, type 2 diabetes mellitus, dyslipidaemia.
